# Impact of baseline clinical features on outcomes of nebulized glycopyrrolate therapy in COPD

**DOI:** 10.1038/s41533-021-00255-7

**Published:** 2021-10-07

**Authors:** Donald P. Tashkin, Xiaoli Niu, Sanjay Sharma, Shahin Sanjar

**Affiliations:** 1 Division of Pulmonary and Critical Care Medicine, David Geffen School of Medicine at UCLA Health Sciences, Los Angeles, CA USA; 2 Sunovion Pharmaceuticals Inc, Marlborough, MA USA

**Keywords:** Therapeutics, Chronic obstructive pulmonary disease

## Abstract

Inhaled bronchodilators are central for the treatment of chronic obstructive pulmonary disease (COPD), as they can provide symptom relief and reduce the frequency and severity of exacerbations while improving health status and exercise tolerance. In 2017, glycopyrrolate (GLY) delivered via the eFlow® closed system (CS) nebulizer (nebulized GLY; 25 µg twice daily), was approved by the US Food and Drug Administration for maintenance treatment of moderate-to-very-severe COPD. This approval was based largely on results from the replicate, placebo-controlled, Phase III clinical trials- GOLDEN 3 and 4. In this review, we summarize key findings from secondary analyses of the GOLDEN 3 and 4 studies, and provide a comprehensive overview that may assist both pulmonologists and primary-care providers in their treatment decisions. Comorbidities are common among patients with COPD in clinical practice and may impact bronchodilator efficacy. This review highlights outcomes among subpopulations of patients with comorbidities (e.g., anxiety/depression, cardiovascular disease), and their impact on the efficacy of nebulized GLY. In addition, the efficacy and safety of nebulized GLY across various demographics (e.g., age, gender) and baseline disease characteristics (e.g., disease severity, rescue medication use) are discussed. Real-world outcomes with nebulized GLY, including device satisfaction, healthcare resource utilization, and exacerbations, are also presented. These secondary analyses and real-world data complement the primary results with nebulized GLY from Phase III studies and support the need for the inclusion of patients representative of real-world clinical practice in RCTs. In addition, these data suggest that RCTs for COPD therapies should be complemented with real-world observational studies.

## Introduction

Chronic obstructive pulmonary disease (COPD) is a progressive disease characterized by persistent respiratory symptoms and airflow limitation^1^. The main causes of COPD are smoking or significant exposure to noxious particles (e.g., environmental inhaled particulates, air pollution), although genetic factors, aging, and abnormal lung development may also play a role. COPD is considered the third leading cause of death in the US, with an estimated 15.7 million adults (6.4% of adults in the US) diagnosed with COPD; however, COPD remains highly underdiagnosed, and the actual number of patients may be higher^1,2^. COPD also represents a substantial socioeconomic burden (US: $50 billion annual direct and indirect costs in 2010)^3^.

Treatment of patients with COPD is based on clinical parameters including symptom burden, spirometry, and exacerbation history. The Global Initiative for Chronic Obstructive Lung Disease (GOLD) report provides recommendations for the treatment of patients with COPD to best target their symptoms and exacerbation risk^1^. Inhaled bronchodilators are the cornerstone of COPD treatment, and provide symptom relief, reduce the frequency and severity of exacerbations, and improve exercise tolerance and health status^1^. There are two general classes of bronchodilators-β_2_-agonists and anticholinergics (or muscarinic antagonists), which are further subdivided into short-acting (~4–6 h) and long-acting (≥12 h) medications based on the duration of their action^1^. Additional use of inhaled corticosteroids (ICS) is based on exacerbation frequency, with growing evidence suggesting that blood eosinophil counts may be used as a biomarker to support ICS use^1^. Pulmonary delivery of bronchodilators requires handheld devices (e.g., pressurized metered-dose inhalers, dry powder inhalers (DPI), soft mist inhalers) or nebulizer systems. To ensure optimal management of COPD, GOLD recommends personalized selection of both bronchodilators and their delivery systems based on individual patient characteristics^1^.

The approval of drugs and devices for the treatment of patients with COPD is based on randomized clinical trials (RCTs), the “gold standard” to assess the efficacy and safety of any therapeutic agent. However, RCTs utilize varying restrictive inclusion (e.g., age, disease severity) and exclusion (e.g., comorbidities, concomitant medications) criteria, leading to variabilities between patients in RCTs and those in the real world. RCTs for COPD regularly include a higher proportion of younger participants, male patients, and those with few comorbidities^4,5^. This contrasts with the real-world COPD population, where there is a higher prevalence of older patients, females, and patients with multiple comorbidities^1,2^. When key selection criteria from 31 RCTs of inhaled long-acting bronchodilators were applied to >36,000 patients with COPD in a real-world setting, only ~25% of patients were eligible for inclusion in these studies^4^. These data suggest major gaps in RCT design that impact the generalization of outcomes to real-world patients, highlighting the need for more permissive recruitment criteria to align with real-world patient populations.

**Fig. 1:**
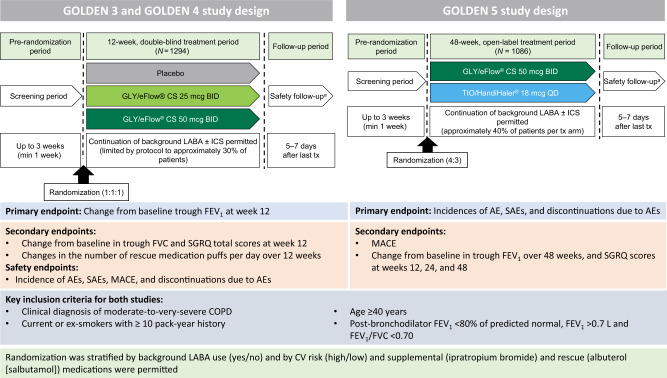
GOLDEN 3, GOLDEN 4, and GOLDEN 5 study designs. ^a^SAEs were monitored for 30 days after the last dose of study treatment. *AE* adverse event, *BID* twice daily, *CS* closed system, *CV* cardiovascular, *FEV*_*1*_ forced expiratory volume in 1 s, *FVC* forced vital capacity, *GLY* nebulized glycopyrrolate, *ICS* inhaled corticosteroids, *LABA* long-acting β_2_-agonist, *MACE* major adverse cardiac event, min minimum, *QD* once daily, *TIO* tiotropium, *SAE* serious AE, *tx* treatment, *SGRQ* St. George’s Respiratory Questionnaire.

In December 2017, glycopyrrolate (GLY) inhalation solution (Lonhala^®^ [Sunovion Pharmaceuticals, Inc., Marlborough, MA, USA]) 25 μg twice daily (BID) delivered via the eFlow^®^ Closed System (CS) Nebulizer (Magnair^®^ [PARI Pharma GmbH, Starnberg, Germany]) was approved by the US Food and Drug Administration (FDA) for the long-term maintenance treatment of airflow obstruction in patients with moderate-to-very-severe COPD (referred henceforth as ‘nebulized GLY’)^6^. This was based, in part, on the results of two Phase III studies (Glycopyrrolate for Obstructive Lung Disease via Electronic Nebulizer [GOLDEN 3; NCT02347761] and GOLDEN 4 [NCT02347774]; Fig. 1)^7^.

Although many clinical trials in COPD include patients with limited real-world applicability, the GOLDEN 3 and 4 studies were prospectively designed to include patients who had pre-existing cardiovascular disease (CVD), cardiovascular (CV) risk factors, and background long-acting β_2_-agonist (LABA) therapies; hence, these studies offer insight into the safety and efficacy of nebulized GLY in subgroups of patients who are typically not represented in RCTs. The objective of this review is to provide a comprehensive overview of treatment outcomes with nebulized GLY in patients representative of the real-world COPD population, which may impact treatment decisions among pulmonologists and primary-care providers. In particular, we highlight data with nebulized GLY in patients with comorbidities, which are common in COPD patients in clinical practice and can affect morbidity, quality of life, and mortality.

### Phase III studies of nebulized GLY

Three Phase III studies were conducted to evaluate the safety and efficacy of GLY 25 µg and GLY 50 µg BID doses, delivered via the eFlow^®^ CS nebulizer. GOLDEN 3 and 4 were replicated, randomized, multi-center, placebo-controlled, double-blind studies, in 1293 patients with moderate-to-very-severe COPD. Patients were randomized to receive either placebo or GLY (25 or 50 µg BID), via the eFlow^®^ CS nebulizer for 12 weeks^7^. The primary endpoint of both studies was the change from baseline in trough forced expiratory volume in 1 s (FEV_1_) at week 12 (trough FEV_1_ is the average of FEV_1_ values collected at the end of the dosing interval at each clinic visit). GOLDEN 5 was a 48-week, randomized, open-label, active-controlled study in 1086 patients that assessed the long-term safety and tolerability of GLY 50 µg BID (via the eFlow^®^ CS nebulizer) compared with tiotropium (TIO) 18 µg QD (via Handihaler^®^ DPI)^8^. The recruitment criteria for GOLDEN 5 were identical to those of GOLDEN 3 and 4; primary endpoints were the incidences of adverse events (AE), serious AEs (SAEs), and discontinuations owing to AEs. Key details for these three trials are shown in Fig. 1. In GOLDEN 3 and 4, nebulized GLY resulted in significant improvements from baseline in trough FEV_1_ and St. George’s Respiratory Questionnaire (SGRQ) total scores compared with placebo, at 12 weeks. In GOLDEN 5, treatment with GLY 50 µg BID, resulted in sustained improvements from baseline in FEV_1_ compared with TIO 18 µg once daily (QD), at 48 weeks. Key efficacy and safety results from these studies are summarized in Table 1. Further, analysis of health-related quality of life (HRQoL) data from the three Phase III studies showed significant improvements from baseline with GLY relative to placebo in SGRQ total and component scores^9^. In GOLDEN 3 and 4, a higher proportion of patients in the GLY 25 µg BID group showed ≥4-unit improvements (representing minimally clinically important differences) in SGRQ total scores (SGRQ responders), compared with placebo (GLY: 47%; placebo: 35%); for GOLDEN 5, SGRQ responder rates at week 48 were similar between GLY 50 µg BID and TIO 18 µg QD groups.

**Table 1:** Overview of key efficacy and safety results from the GOLDEN 3, GOLDEN 4, and GOLDEN 5 Phase III studies of nebulized GLY.

Study	Key efficacy and safety outcomes
GOLDEN 3 (Kerwin EM et al., 2017)^7^	Efficacy:
	• Significant improvements from baseline in trough FEV_1_ compared with placebo at week 12: ‒ LS mean placebo-adjusted change from baseline:105 mL and 126 mL, for the GLY 25 µg and 50 µg BID doses, respectively (*p* < 0.001). •Placebo-adjusted changes from baseline in SGRQ scores were significant only for the 25 µg BID group at week 12: ‒ LS mean difference of –3.072 (*p* < 0.05) and –1.848 (*p* > 0.05), for the GLY 25 µg BID and 50 µg BID groups, respectively. • Improvements in SGRQ responder rates of 39.7%, 49.7%, and 44.1%, with placebo, GLY 25 µg BID, and GLY 50 µg BID, respectively.
	Safety:
	• AEs: 52%, 40%, and 48%, with placebo, GLY 25 μg, and GLY 50 μg BID, respectively. • AEs leading to treatment discontinuation: 10%, 3%, and 4% with placebo, GLY 25 μg, and GLY 50 μg BID, respectively.
GOLDEN 4 (Kerwin EM et al., 2017)^7^	Efficacy:
	• Significant improvements from baseline in trough FEV_1_ compared with placebo at Week 12: ‒ LS mean placebo-adjusted change from baseline: 84 mL and 82 mL, for the GLY 25 µg and 50 µg BID doses, respectively (*p* < 0.001). • Significant improvements from baseline (*p* < 0.01) in placebo-adjusted SGRQ total scores in both GLY treatment groups at Week 12: ‒ LS mean difference of –3.59 and –3.56, for the GLY 25 µg BID and 50 µg BID groups, respectively. • Improvements in SGRQ responder rates of 29.6%, 43.7%, and 39.4%, with placebo, GLY 25 µg BID, and GLY 50 µg BID, respectively.
	Safety:
	• AEs: 52%, 47%, and 53%, with placebo, GLY 25 μg, and GLY 50 μg BID, respectively. • AEs leading to treatment discontinuation: 9%, 7%, and 4% with placebo, GLY 25 μg, and GLY 50 μg BID, respectively.
GOLDEN 5 (Ferguson GT et al., 2017)^8^	Efficacy:
	• Sustained improvements in FEV_1_ with GLY 50 µg BID at 48 weeks: ‒ LS mean change from baseline in trough FEV_1_ was 102 mL and 93 mL, with GLY 50 µg BID and TIO 18 µg QD, respectively. • Improvements were also observed in SGRQ total scores in both treatment groups at 48 weeks: ‒ LS mean changes from baseline were –3.07 and –4.08, with GLY 50 µg BID and TIO 18 µg QD, respectively.
	Safety:
	• AEs: GLY 50 μg BID: 69.4%; TIO 18 μg QD: 67.0%. • SAEs: GLY 50 μg BID: 12.3%; TIO 18 μg QD: 10.5%. • AEs leading to treatment discontinuation: GLY 50 μg BID: 10%; TIO 18 μg QD: 2.8%.

AE adverse event, BID twice daily, FEV_1_ forced expiratory volume in 1 s, GLY nebulized glycopyrrolate, LS least squares, QD once daily, SAE serious AE, SGRQ St. George’s Respiratory Questionnaire

Nebulized GLY was generally well tolerated in the short-term (12-week) and long-term (48-week) studies, with the most commonly reported AEs being cough and worsening of COPD^7^. In GOLDEN 3 and 4, patients receiving either dose of GLY had lower incidences of AEs compared with placebo (Table 1). The incidence of SAEs was low (<5%), whereas treatment discontinuation due to AEs was highest with placebo. In GOLDEN 5, the incidence of AEs and SAEs over 48 weeks was similar between the GLY 50 μg BID and TIO 18 μg QD treatment groups^8^. The incidence of major adverse cardiac events (non-fatal myocardial infarction, non-fatal stroke, and CV death), was higher in the TIO 18 μg QD arm compared with the GLY 50 μg BID arm (20.3 vs. 6.4 per 1000 patient-years). The higher rate of treatment discontinuation owing to AEs in the GLY 50 μg BID group may be attributed to greater errors in breathing technique with a nebulizer, which were likely owing to a lack of training.

Clinically important deterioration (CID) is a composite endpoint to measure worsening in COPD and evaluate the efficacy of bronchodilators in clinical trials^10^. Patients were classified as having experienced CID if any of the following had occurred: (1) a ≥100 mL decrease from baseline in post-bronchodilator trough FEV_1_; (2) ≥4-unit increase from baseline in SGRQ total scores (indicative of worsening COPD); or (3) a moderate or severe healthcare resource utilization (HCRU)-related exacerbation^11^. At 12 weeks, nebulized GLY led to fewer CID events compared with placebo (GLY 25 µg: 34%; placebo: 51%), in the GOLDEN 3 and 4 studies^12^. Nebulized GLY significantly reduced the risk of any CID by 50% compared with placebo, reducing the risk of ≥100 mL decrease from baseline in post-bronchodilator trough FEV_1_ by ~60%, and the risk of ≥4-unit increase from baseline in SGRQ total scores by 48% (*p* < 0.05 for both). The risk of CID was significantly lower with GLY 25 µg BID regardless of age, smoking status, or baseline COPD severity.

### Secondary analyses of the GOLDEN 3 and 4 studies

Several secondary analyses of the GOLDEN 3 and 4 studies were conducted to examine the effect of demographics (e.g., gender, age), baseline disease characteristics (e.g., disease severity, background LABA use, rescue medication use), and comorbidities on the efficacy and safety of nebulized GLY in subpopulations of patients with moderate-to-very-severe COPD. These provide insight into the outcomes of treatment with nebulized GLY in patients similar to those in clinical settings.

**Fig. 2:**
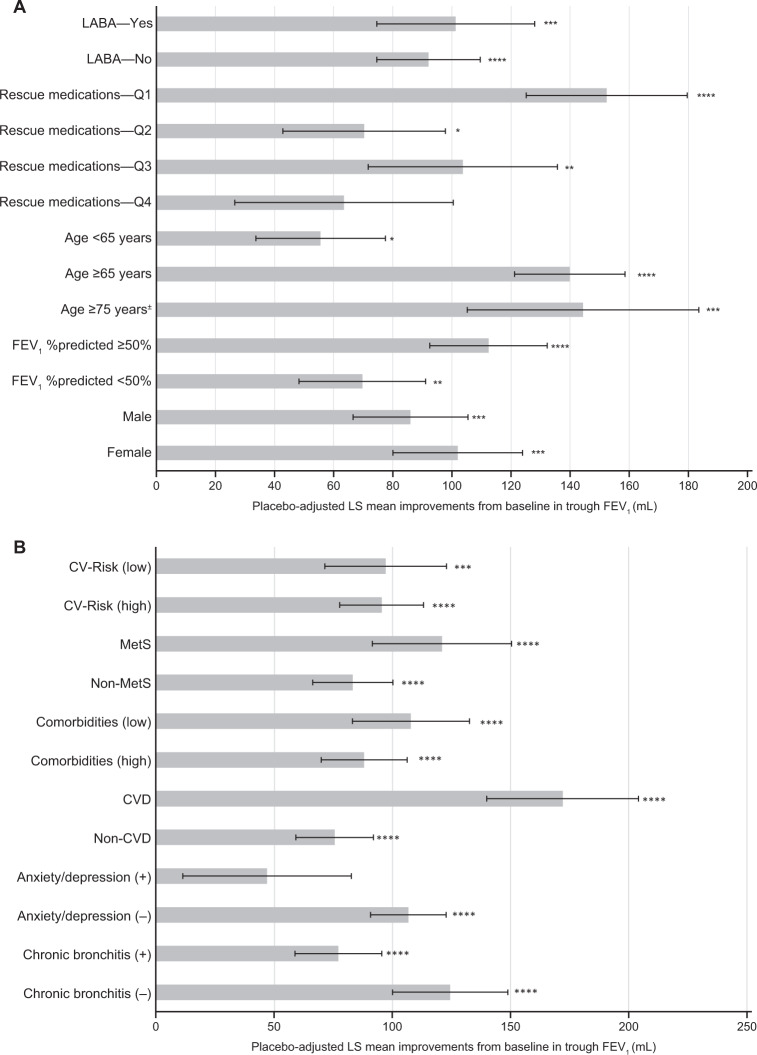
Placebo-adjusted improvements from baseline in trough FEV1 (mL). Analysis by **A** baseline demographics and disease severity, and **B** comorbidities and chronic bronchitis at baseline, at 12 weeks. *p < 0.05; **p < 0.01; ***p < 0.001; ****p < 0.0001 versus placebo, analyzed using a mixed model for repeated measures. Only placebo and GLY 25 µg dose from the GOLDEN 3 and 4 studies are shown. ^±^Subset of the ≥65 years group. *CV* cardiovascular, *CVD* cardiovascular disease, *FEV*_*1*_ forced expiratory volume in 1 s, *GLY* nebulized glycopyrrolate, *LABA* long-acting β_2_-agonist, *LS* least squares, *MetS* metabolic syndrome, *Q* quarter.

**Fig. 3:**
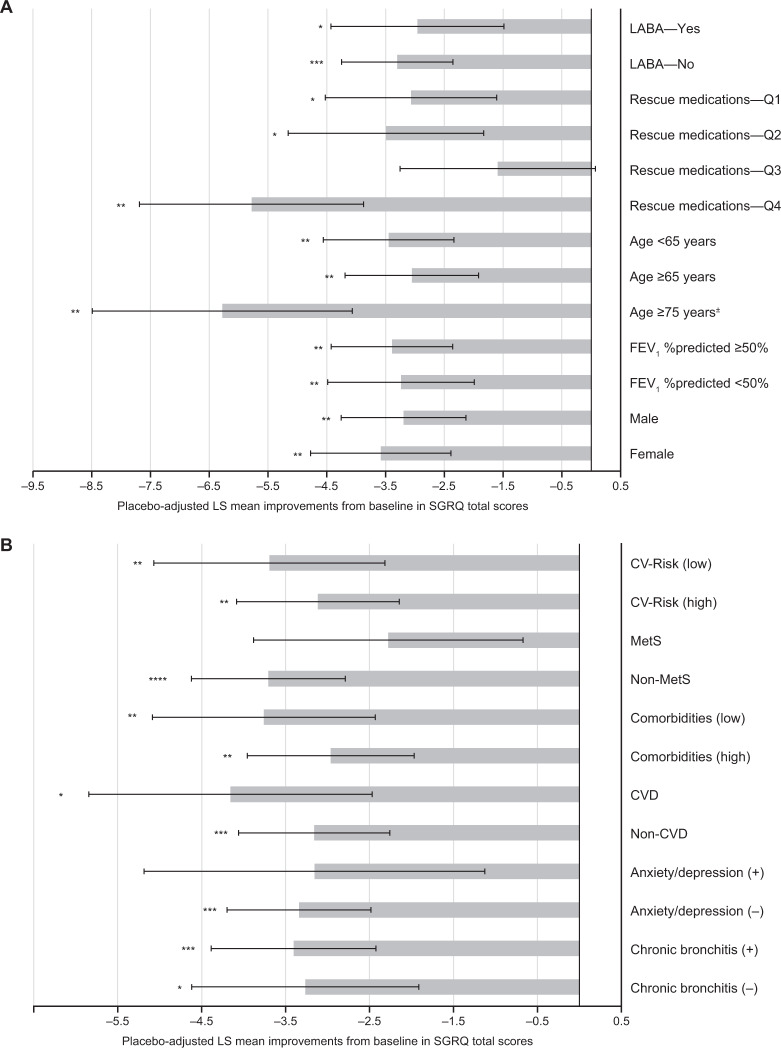
Placebo-adjusted improvements from baseline in SGRQ total scores. Analysis by **A** baseline demographics and disease severity, and **B** comorbidities and chronic bronchitis at baseline, at 12 weeks. *p < 0.05; **p < 0.01; ***p < 0.001; ****p < 0.0001 versus placebo, analyzed by ANCOVA. Only placebo and GLY 25 µg dose from the GOLDEN 3 and 4 studies are shown. ^±^Subset of the ≥65 years group. *CV* cardiovascular, *CVD* cardiovascular disease, *FEV*_*1*_ forced expiratory volume in 1 s, *GLY* nebulized glycopyrrolate, *LABA* long-acting β_2_-agonist, *LS* least squares, *MetS* metabolic syndrome, *Q* quarter, *SGRQ* St. George’s Respiratory Questionnaire.

**Fig. 4:**
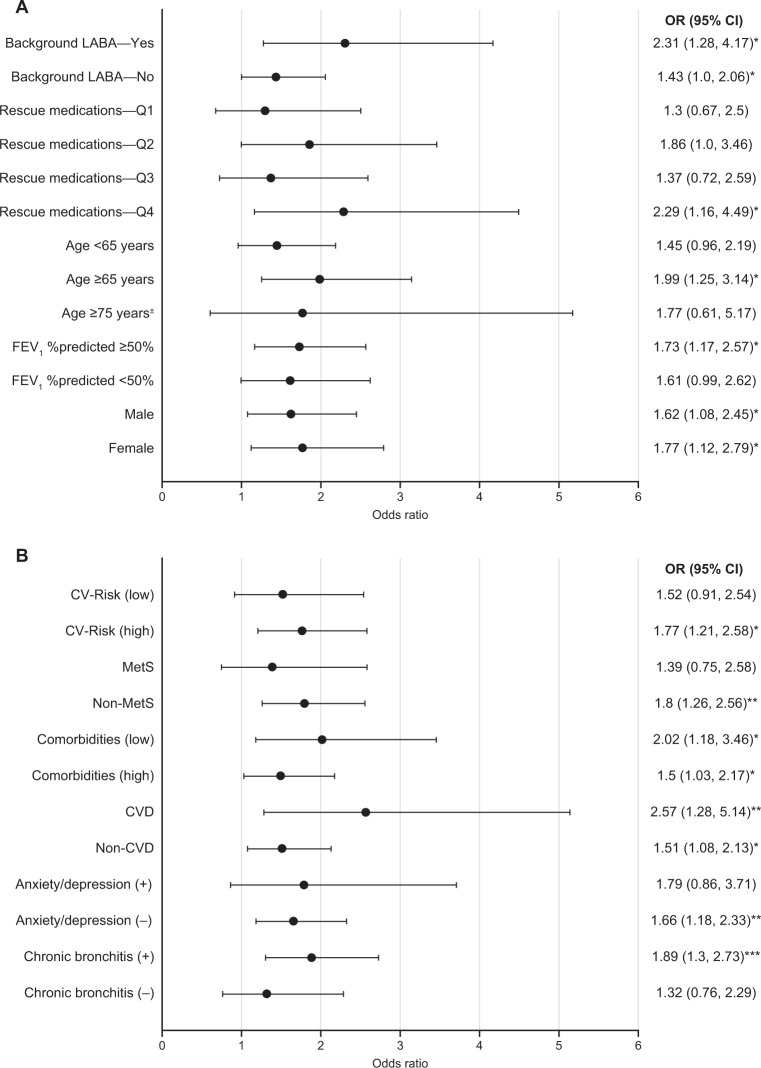
SGRQ responder rates with GLY compared with placebo. Analysis by **A** baseline demographics and disease severity, and **B** comorbidities and chronic bronchitis at baseline. *p < 0.05; **p < 0.01; ***p < 0.001 versus placebo, analyzed using a logistic regression model. *CI* confidence interval, *CV* cardiovascular, *CVD* cardiovascular disease, *FEV*_*1*_ forced expiratory volume in 1 s, *GLY* nebulized glycopyrrolate, *LABA* long-acting β_2_-agonist, *LS* least squares, *MetS* metabolic syndrome, *OR* odds ratio, *Q* quartile, *SGRQ* St. George’s respiratory questionnaire.

In recent years, COPD has been shown to affect more women than men; women represent 58% of the total patients diagnosed with COPD and are 37% more likely to have COPD than men. The disease trajectory also varies between the genders, and distinct differences in the symptoms and progression of COPD are often noted in women compared with men, which may impact their responses to bronchodilator therapy^13,14^. However, women are routinely under-represented in clinical trials for COPD with most including 75–77% male patients^15–18^, while treatment recommendations do not consider gender as a factor that may impact treatment choice. In GOLDEN 3 and 4, women represented ~45% of patients^7^. At Week 12, nebulized GLY significantly improved lung function and SGRQ total scores, irrespective of gender (Figs. 2A, 3A, and Table 2)^19^. Further, the odds of being an SGRQ responder were significantly higher with GLY compared with placebo, regardless of gender; the odds of being an Exacerbations of Chronic Pulmonary Disease Tool-Respiratory Symptoms (EXACT-RS) responder (≥2-unit reduction in total score) were significantly higher with GLY compared with placebo only in men (Fig. 4A and Supplementary Fig. 1). These data support the efficacy of nebulized GLY in patients with COPD, independent of gender.

**Table 2:** Overview of secondary analyses of the GOLDEN 3 and 4 studies^†^, by demographic and disease characteristics at baseline.

Study	Patients	Placebo-adjusted improvements in LS mean change from baseline with GLY				
Background LABA (Kerwin EM et al., 2018)^27^	LABA-yes: *n* = 267; LABA-no: *n* = 594	FEV_1_	LABA-yes: 101 mL***		LABA-yes: −2.957*	
		SGRQ	LABA-yes: −2.957*		LABA-no: −3.301***	
		E-RS	LABA-yes: −0.91		LABA-no: −1.165**	
Rescue medications^‡^ (Donohue JF et al., 2020)^29^	Q1: *n* = 188; Q2: *n* = 206; Q3: *n* = 198; Q4: *n* = 189	FEV_1_	Q1: 152 mL***	Q2: 70 mL*	Q3: 104 mL**	Q4: 64 mL
		SGRQ	Q1: −3.07*	Q2: −3.49*	Q3: −1.59	Q4: −5.78**
		EXACT-RS	Q1: −1.23*	Q2: −1.13	Q3: −0.72	Q4: −1.81
Age (Ohar JA et al. 2019)^25^	Age <65: *n* = 451; Age ≥65: *n* = 410; Age ≥75^§^: *n* = 88	FEV_1_	<65 years: 56 mL*		≥65 years: 140 mL*** ≥75^§^ years: 144 mL***	
		SGRQ	<65 years: −3.447**		≥65 years: −3.053** ≥75^§^ years: −6.278**	
Disease severity (Ohar JA et al., 2019)^25^	FEV_1_%predicted ≥50%: *n* = 498;	FEV_1_	FEV_1_ %predicted	≥50%: 112 mL***	FEV_1_ %predicted	<50%: 70 mL**
	FEV_1_ %predicted *n* = 362	SGRQ	FEV_1_ %predicted	≥50%: −3.392**	FEV_1_ %predicted	<50%: −3.237**
Gender (Ohar JA et al., 2020)^19^	Male: *n* = 477 Female: *n* = 384	FEV_1_	Male: 86 mL***		Female: 102 mL***	
		SGRQ	Male: −3.19**		Female: −3.58**	
		EXACT-RS	Male: −0.82		Female: −1.48**	

*p < 0.05; **p < 0.01; ***p < 0.001.

^†^Only placebo and GLY 25 µg BID dose from the GOLDEN 3 and 4 studies are shown.

^‡^Patients were divided into four quartile subgroups based on rescue medication use (<25, 25–<50, 50–<75, and >75 percentile of rescue medication use).

^§^Subset of the ≥65 years group.

*BID* twice daily, *E-RS* evaluating respiratory symptoms in COPD, *EXACT-RS* exacerbations of chronic pulmonary disease tool-respiratory symptoms, *FEV*_*1*_ forced expiratory volume in 1 s, *GLY* nebulized glycopyrrolate, *LABA* long-acting β_2_-agonist, *LS* least squares, *Q* quarter, *SGRQ* St. George’s Respiratory Questionnaire.

It is estimated that ~35% of patients diagnosed with COPD in the US are ≥65 years of age^20^. Age is considered to be a risk factor for COPD, with a progressive decline in lung function often observed with aging^1,21,22^. Physiological changes to the lungs (e.g., reduction in strength of the respiratory muscles, change in thorax shape due to osteoporosis) and cognitive impairments (e.g., Alzheimer’s disease, dementia) due to aging, may impair bronchodilator efficacy in elderly patients with COPD^23,24^. Nebulizers may be advantageous in this population, as drug delivery is through normal tidal breathing, in contrast to the special breathing techniques required with handheld inhalers; therefore, nebulizers are suited for patients with physical/cognitive disabilities^23^. In GOLDEN 3 and 4, nebulized GLY significantly improved lung function and SGRQ total scores, irrespective of age (Figs. 2A and 3A)^25^. At week 12, greater improvements from baseline in placebo-adjusted FEV_1_ with nebulized GLY were observed among older versus younger patients (age ≥65 years: 140 mL; age <65 years: 56 mL; Table 2). Although the odds of being an SGRQ responder were numerically higher with GLY compared with placebo irrespective of age, significant improvements were observed only among patients ≥65 years of age (Fig. 4A). These outcomes demonstrate the efficacy of nebulized GLY in patients with COPD independent of age, including those aged ≥75 years.

Approximately 30% of patients have severe or very-severe COPD (i.e., FEV_1_ < 50% of predicted normal) at the time of diagnosis^26^. This impacts treatment efficacy in COPD because bronchodilator responses are known to decline with increasing disease severity; patients with severe/very-severe COPD (GOLD III and IV) show large declines in FEV_1_ bronchodilator responses over 4 years, compared with those with mild/moderate disease (GOLD I/II)^21^. To assess the impact of disease severity on the efficacy and safety of nebulized GLY, patients from the GOLDEN 3 and 4 studies were grouped by their baseline post-bronchodilator FEV_1_ %predicted (≥50%: mild-to-moderate; <50%: severe-to-very-severe)^25^. At 12 weeks, nebulized GLY significantly improved lung function and SGRQ total scores, irrespective of disease severity (Figs. 2A, 3A, and Table 2). Although the odds of being an SGRQ responder were numerically higher with GLY compared with placebo in both FEV_1_ groups, significant improvements were observed only among patients in the FEV_1_ %predicted ≥50% group (Fig. 4A). These results highlight the efficacy of nebulized GLY in patients with COPD, irrespective of baseline disease severity.

Combination therapies (e.g., long-acting muscarinic antagonists + LABA ± ICS) are often utilized for the management of moderate-to-very-severe COPD^1^. Patients from the GOLDEN 3 and 4 studies were grouped by background LABA (±ICS) use, and the efficacy and safety of nebulized GLY were examined^27^. Following 12 weeks of treatment, nebulized GLY significantly improved lung function and SGRQ total scores, regardless of background LABA use (Figs. 2A, 3A, and Table 2). The odds of being an SGRQ responder (≥4-unit reduction in total scores) was significantly greater with GLY compared with placebo, regardless of background LABA use (Fig. 4A). These results demonstrate the efficacy of nebulized GLY, independent of background LABA ± ICS use.

Rescue medications (e.g., short-acting bronchodilators) are commonly used in COPD management, and the frequency of their use often correlates with increased disease severity and exacerbations^1,28^. To understand the impact of baseline rescue medication use on the efficacy of nebulized GLY, patients were divided into four quartile groups (Q1: <25 percentile; Q2: 25–<50; Q3: 50–<75; Q4: ≥75 percentile of rescue medication use)^29^. At 12 weeks, significant improvements from baseline in FEV_1_ were observed with GLY in all rescue medication subgroups except Q4 (Fig. 2A and Table 2); significant improvements in SGRQ total scores were noted with GLY compared with placebo in all baseline rescue medication subgroups except Q3 (Fig. 3A). The odds of being an SGRQ and EXACT-RS responder were numerically higher with GLY compared with placebo in all baseline rescue medication subgroups, but only significant in the Q4 subgroup (Fig. 4A and Supplementary Fig. 1). These results demonstrated that treatment with nebulized GLY improved lung function and symptom scores, regardless of baseline rescue medication use.

In clinical practice, patients with COPD often present with multiple comorbidities, and these can have an impact on both COPD progression and the effectiveness of bronchodilator therapy^30^. The GOLDEN 3 and 4 studies allowed the recruitment of patients with baseline comorbidities, thereby providing insight into the efficacy and safety of nebulized GLY in these patients.

#### Overall comorbidity prevalence

COPD progression, exacerbation frequency, and mortality are often impacted by comorbidities; the most prevalent comorbidities in patients with COPD include CVD, lung cancer, and diabetes^31,32^. A simple comorbidity count method was developed to identify and characterize comorbidities in COPD; analysis of two large COPD cohorts, COPDGene^®^ and SPIROMICS, using this method showed that determining a comorbidity score could provide insight into clinical trial readouts^33^. Using this simple comorbidity count on the pooled GOLDEN 3 and 4 population showed a high prevalence of comorbidities, the most common ones being hypertension, high cholesterol, and osteoarthritis^34^. Nebulized GLY improved lung function and SGRQ scores in individuals with COPD, independent of their comorbidity count (Figs. 2B, 3B, and Table 3). The odds of being an SGRQ responder were significantly higher with GLY compared with placebo, regardless of baseline comorbidity count (Fig. 4B). These results demonstrate that nebulized GLY improved lung function and health status, independent of the presence and number of baseline comorbidities.

**Table 3:** Overview of secondary analyses of the GOLDEN 3 and 4 studies^†^, by comorbidities and chronic bronchitis at baseline.

Study	Patients	Placebo-adjusted improvements in LS mean change from baseline with GLY		
CV Risk (Ferguson GT et al., 2019)^37^	CV risk (low): *n* = 308; CV risk (high): *n* = 553	FEV_1_	CV risk (low): 97 mL***	CV risk (high): 95 mL***
		SGRQ	CV risk (low): –3.69**	CV risk (high): –3.12**
MetS (Carlin B et al., 2020)^40^	MetS: *n* = 217; Non-MetS: *n* = 644	FEV_1_	MetS: 121 mL****	Non-MetS: 83 mL****
		SGRQ	MetS: –2.28	Non-MetS: –3.71****
A/D (Hanania NA et al., 2021)^42^	A/D (+): *n* = 156; A/D (–): *n* = 705	FEV_1_	A/D (+): 47 mL	A/D (–): 107 mL****
		SGRQ	A/D (+): –3.16	A/D (–): –3.34***
Comorbidities (Putcha N et al., 2021)^34^	Comorbidities (low): *n* = 292; Comorbidities (high): *n* = 569	FEV_1_	Comorbidities (low): 108 mL****	Comorbidities (high): 88 mL****
		SGRQ	Comorbidities (low): –3.76**	Comorbidities (high): –2.96**
CVD (Putcha N et al., 2021)^34^	CVD: *n* = 170; Non-CVD: *n* = 691	FEV_1_	CVD: 172 mL****	Non-CVD: 76 mL****
		SGRQ	CVD: –4.16*	Non-CVD: –3.16***
CB (Tashkin DP et al., 2021)^44^	CB: *n* = 554; Non-CB: *n* = 307	FEV_1_	CB: 77 mL**	Non-CB: 124 mL****
		SGRQ	CB: –3.41***	Non-CB: –3.27*

^†^Only placebo and GLY 25 µg BID dose from the GOLDEN 3 and 4 studies are shown.

*p < 0.05; **p < 0.01; ***p < 0.001; ****p < 0.0001 versus placebo.

*A/D* anxiety/depression, *BID* twice daily, *CB* chronic bronchitis, *CV* cardiovascular, *CVD* cardiovascular disease, *FEV*_*1*_ forced expiratory volume in 1 s, *GLY* nebulized glycopyrrolate, *LS* least squares, *MetS* metabolic syndrome, *Q* quarter, *SGRQ* St. George’s Respiratory Questionnaire.

CVD and COPD share a number of risk factors, including smoking, age, and physical inactivity^35^. CVD is prevalent in 28–70% of patients with COPD, and plays a key role in the morbidity and mortality associated with COPD^35^. Owing to the observed cardiac effects of bronchodilators used in COPD therapies, it is important to characterize their efficacy and safety in patients with existing CV risk factors^1,36^. A secondary analysis of the three Phase III GOLDEN studies examined the impact of pre-existing CV risk factors on the safety and efficacy of nebulized GLY^37^. High CV risk was determined based on a history of one or more of the following: ischemic heart disease, cerebrovascular disease, peripheral arterial disease, clinically significant arrhythmia, heart failure, or hypertension. At baseline, 553/861 patients (64%) had a high CV risk. At 12 weeks, nebulized GLY resulted in significant improvements in lung function and SGRQ total scores, regardless of CV risk status at baseline (Figs. 2B, 3B, and Table 3). The odds of being an SGRQ responder were numerically higher with GLY compared with placebo regardless of CV risk at baseline, but only significant in the group with high CV risk (Fig. 4B). Further, nebulized GLY was well tolerated, and had no major safety signals in patients, regardless of their CV risk status (for more details, see Section Combined safety data from subanalyses of GLY/eFlow^®^). These results show that nebulized GLY improved lung function and patient-reported outcomes (PROs) in patients with COPD, independent of CV risk status.

CVD is among the most prevalent comorbidities in patients with COPD^31,32^. A higher prevalence of CVD comorbidities (defined as the presence of any of the following: coronary heart disease, congestive heart failure, or peripheral vascular disease) was noted in patients with higher (>2) versus lower (≤2) comorbidity counts at baseline^34^. Nebulized GLY improved lung function and SGRQ scores in individuals with COPD, independent of their CVD status (Figs. 2B, 3B, and Table 3). The odds of being an SGRQ responder were significantly higher with GLY compared with placebo, regardless of baseline CVD status (Fig. 4B). These results demonstrate that nebulized GLY improved lung function and health status, independent of baseline CVD status.

Metabolic syndrome (MetS) is defined as a clustering of ≥3 CV risk factors, and is twice more common among patients with versus without COPD; the prevalence of MetS in patients with COPD is estimated to be between 20% and 60%^38^. MetS may lead to airflow limitation and may worsen COPD progression^38,39^. Pooled data from the GOLDEN 3 and 4 studies showed ~25% prevalence of MetS at baseline (presence of ≥3 of the following: hypertension, hyperlipidemia, diabetes, and body mass index >30 kg/m^2^)^40^. At 12 weeks, nebulized GLY significantly improved lung function, regardless of MetS at baseline (Fig. 2B and Table 3). Although numerical improvements in SGRQ total scores and responder rates with nebulized GLY were noted in both MetS and non-MetS groups, significant improvements from baseline were only observed in the non-MetS group (Figs. 3B, 4B, and Table 3). These results show that nebulized GLY is an effective treatment option for patients, regardless of their baseline MetS status.

Anxiety and depression (A/D) frequently occur among patients with COPD and have been associated with lower treatment adherence and increased risk of COPD exacerbations and mortality^31,41^. Comorbid A/D was observed in 18% of patients from the pooled GOLDEN 3 and 4 population^42^. Nebulized GLY led to numerical improvements in lung function, SGRQ total scores, and SGRQ responder rates relative to placebo, regardless of A/D status at baseline; however, significant improvements in these parameters were observed only in the A/D (–) group (Figs. 2B, 3B, and Table 3). The lack of significance in improvements with GLY treatment compared with placebo may have been due to difficulties in performing pulmonary function tests, non-compliance to medications, and an increased response to placebo treatment in the A/D (+) group. These results highlight potential differences in response with nebulized GLY based on A/D status, and its impact on the outcomes of clinical trials involving bronchodilators.

Together, these studies emphasize the importance of considering underlying comorbidities when prescribing treatments, and their impact on bronchodilator efficacy in patients with COPD.

Chronic bronchitis (CB) is one of the conditions that comprise COPD and refers to chronic inflammation in the bronchi; CB can accelerate lung function decline, increase COPD exacerbation frequency, and reduce HRQoL^43^. Pooled data from patients from the GOLDEN 3 and 4 studies showed a high prevalence (65%) of SGRQ-defined CB at baseline^44^. At 12 weeks, nebulized GLY showed significant improvements in both FEV_1_ and SGRQ total scores compared with placebo, regardless of CB status at baseline (Figs. 2B, 3B, and Table 3). The odds of being an SGRQ responder were significantly higher with GLY compared with placebo in the CB (+) group only (Fig. 4B). These results showcase the efficacy of nebulized GLY in patients with COPD, independent of CB status.

Nebulized GLY showed a good safety profile across various subgroups of patients, with a low incidence of AEs and SAEs among patients treated with GLY, compared with placebo (Tables 4 and 5). The most commonly reported AEs across these studies were cough, worsening of COPD, and dyspnea, which were consistent with the primary safety results^7^. The incidence of CV events of special interest was low (<5%), among all subgroups of patients, including among those with MetS or high CV risk at baseline (Table 5). Although discontinuation due to AEs was generally low with GLY across these studies (<10%), higher rates were noted in female versus male patients (6.9% vs. 3.7%; Table 4)^19^. In all the subanalyses, the most common AEs leading to discontinuation were cough, worsening of COPD, and dyspnea. Together, secondary analyses of the GOLDEN 3 and 4 studies show that nebulized GLY was generally well tolerated in subgroups of patients, regardless of their age, gender, disease severity, background LABA or baseline rescue medication use, or comorbidities at baseline.

**Table 4:** Adverse events most commonly reported in the secondary analyses of the GOLDEN 3 and 4 studies^†^, by demographic and disease characteristics at baseline.

GLY vs. placebo (%)	Background LABA use (Kerwin EM et al., 2018)^27^	Baseline rescue medication use (Donohue JF et al., 2020)^29^	Age (Ohar JA et al., 2019)^25^	Disease severity (Ohar JA et al., 2019)^25^	Gender (Ohar JA et al., 2020)^19^
AEs	LABA-Yes: 42 vs. 53 LABA-No: 44 vs. 52	Q1: 36 vs. 53 Q2: 45 vs. 53 Q3: 50 vs. 49 Q4: 40 vs. 54	Age <65 years: 41 vs. 51 Age ≥65 years: 47 vs. 53 Age ≥75 years^§^: 43 vs. 46	FEV_1_ %predicted <50%: 40 vs. 49 FEV_1_ %predicted ≥50%: 46 vs. 55	Male: 40 vs. 51 Female: 48 vs. 54
SAEs	LABA-Yes: 3.0 vs. 6.8 LABA-No: 3.0 vs. 5.0	Q1: 3.3 vs. 5.2 Q2: 2.2 vs. 4.4 Q3: 3.6 vs. 4.7 Q4: 3.1 vs. 7.5	Age <65 years: 2.6 vs. 4.1 Age ≥65 years: 3.5 vs. 7.1 Age ≥75 years^§^: 0 vs. 7.3	FEV_1_ %predicted <50%: 3.8 vs. 6.8 FEV_1_ %predicted ≥50%: 2.4 vs. 4.7	Male: 3.7 vs. 6.0 Female: 3.2 vs. 5.1
CV Events	LABA-Yes: 0 vs. 0 LABA-No: 0 vs. 0.7	N/A	Age <65 years: 2.2 vs. 1.8 Age ≥65 years: 1.0 vs. 3.3 Age ≥75 years^§^: 4.3 vs. 4.9	FEV_1_ %predicted <50%: 1.1 vs. 2.3 FEV_1_ %predicted ≥50%: 2.0 vs. 2.8	N/A
AEs leading to discontinuation	LABA-Yes: 4.4 vs. 9.1 LABA-No: 5.4 vs. 9.4	N/A	Age <65 years: 3.9 vs. 5.9 Age ≥65 years: 6.5 vs. 12.9 Age ≥75 years^§^: 6.4 vs. 9.8	FEV_1_ %predicted <50%: 4.9 vs. 10.7 FEV_1_ %predicted ≥50%: 5.3 vs. 8.3	Male: 3.7 vs. 8.9 Female: 6.9 vs. 9.7

^†^Only placebo and GLY 25 µg BID dose from the GOLDEN 3 and 4 studies are shown.

^¶^Patients were divided into four quartile subgroups based on rescue medication use (<25, 25–<50, 50–<75, and >75 percentile of rescue medication use).

^§^Subset of the ≥65 years group.

*AE* adverse event, *BID* twice daily, *CV* cardiovascular, *FEV*_*1*_ forced expiratory volume in 1 s, *GLY* nebulized glycopyrrolate, *LABA* long-acting β_2_-agonist, *N/A* not analyzed, *Q* quarter, *SAE* serious AE.

**Table 5:** Adverse events most commonly reported in the secondary analyses of the GOLDEN 3 and 4 studies^†^, by comorbidities and chronic bronchitis at baseline.

GLY vs. placebo (%)	CV risk (Ferguson GT et al., 2019)^37^	MetS (Carlin B et al., 2020)^40^	A/D (Hanania NA et al., 2021)^42^	Comorbidities (Putcha N et al., 2021)^34^	CVD (Putcha N et al., 2021)^34^	CB (Tashkin DP et al., 2021)^44^
AEs	CV risk (high): 41 vs. 53 CV risk (low): 47 vs. 52	MetS: 46 vs. 52 Non-MetS: 43 vs. 53	A/D(+): 54 vs. 67 A/D(–): 41 vs. 49	Comorbidities (low): 43 vs. 46 Comorbidities (high): 44 vs. 55	CVD: 38 vs. 53 Non-CVD: 45 vs. 52	CB (+): 42 vs. 51 CB (–): 45 vs. 55
SAEs	CV risk (high): 4.4 vs. 7.2 CV risk (low): 0.6 vs. 2.6	MetS: 5.0 vs. 6.9 Non-MetS: 2.4 vs. 5.1	A/D(+): 7.4 vs. 9.3 A/D(–): 2.0 vs. 4.8	Comorbidities (low): 2.6 vs. 4.3 Comorbidities (high): 3.2 vs. 6.2	CVD: 2.6 vs. 9.7 Non-CVD: 3.1 vs. 4.5	CB (+): 3.2 vs. 5.9 CB (–): 2.7 vs. 5.1
CV Events	CV risk (high): 1.1 vs. 3.6 CV risk (low): 2.6 vs. 0.7	Cardiac disorders: MetS: 1.0 vs. 4.3 Non-Mets: 1.5 vs. 1.6	N/A	N/A	CVD: 3.9 vs. 4.3 Non-CVD: 1.1 vs. 2.1	N/A
		Hypertension: MetS: 2.0 vs. 2.6 Non-MetS: 0.9 vs. 1.3				
AEs leading to discontinuation	CV risk (high): 6.2 vs. 9.0 CV risk (low): 3.2 vs. 9.9	N/A	N/A	Comorbidities (low): 5.2 vs. 10.1 Comorbidities (high): 5.4 vs. 8.6	CVD: 3.9 vs. 5.4 Non-CVD: 5.4 vs. 10.4	CB (+): 5.0 vs. 7.7 CB (–): 5.3 vs. 12.1

^†^Only placebo and GLY 25 µg BID dose from the GOLDEN 3 and 4 studies are shown.

*A/D* anxiety/depression, *AE* adverse event, *BID* twice daily, *CB* chronic bronchitis, *CV* cardiovascular, *CVD* cardiovascular disease, *GLY* nebulized glycopyrrolate, *MetS* metabolic syndrome, *N/A* not analyzed, *SAE* serious AE.

### Device satisfaction, HCRU, and exacerbations in real-world patients using GLY/eFlow^®^ CS

Patient satisfaction and confidence with inhalation devices in COPD are associated with higher treatment compliance, adherence, and better treatment outcomes^45,46^. Patient satisfaction with GLY/eFlow^®^ CS nebulizer was assessed in a cross-sectional survey among patients with COPD who were using the GLY/eFlow^®^ CS nebulizer in real-world settings. Out of 66 patients who completed the survey, over 90% of patients were “satisfied”/“very satisfied” with the device. On a Likert scale of 1 (“I don’t like it”) to 7 (“I like it a lot”), mean scores were at least 5.9 for portability, ease of cleaning, short administration time, and silence of operation. Overall, results from this real-world study showed a high degree of patient satisfaction and confidence in using GLY/eFlow^®^ CS nebulizer^47^.

A retrospective claims database analysis compared medication use, HCRU, and exacerbations during the 6-month pre-index period and 6-months after initiating treatment with GLY/eFlow^®^ CS nebulizer among 767 patients with COPD^48^. This study demonstrated a significant decrease (*p* < 0.05) in use of COPD-related medications (e.g., antibiotics: 67% vs. 71%; oral corticosteroids: 62% vs. 69%; fixed-dose short-acting muscarinic antagonists/short-acting β_2_-agonists: 26% vs. 33%), lower COPD-related outpatient physician’s office visits, and fewer exacerbations in the 6-month follow-up period, compared with the pre-index period. Patients initiating GLY/eFlow^®^ also had significantly lower all-cause hospital admissions (8% vs. 13%; *p* < 0.05) and shorter hospital stays (mean 1.9 vs. 3.6 days; *p* < 0.05) in the follow-up period, compared with the pre-index period. These real-world findings suggest that nebulized GLY may provide improved symptom control and can reduce the treatment burden in COPD.

## Discussion

The efficacy and safety of nebulized GLY for treatment of moderate-to-very-severe COPD were demonstrated in the Phase III GOLDEN 3 and 4 studies^7^. These secondary analyses of the GOLDEN 3 and 4 studies showed similar efficacy and safety across a variety of clinical features and support the use of nebulized GLY in specific subgroups of patients, regardless of their age, gender, or disease severity, and presence of comorbidities at baseline. These findings are encouraging from a clinical management perspective and highlight the broad applicability of nebulized GLY across COPD patients. These analyses also provide insight into the benefits of nebulized GLY in patients representative of a real-world COPD patient population, which includes a higher proportion of women, older patients, and patients with comorbidities; these subpopulations are poorly represented in COPD RCTs. The outcomes from the secondary analyses are further supported by real-world evidence showing high (~90%) patient satisfaction with GLY/eFlow^®^ CS^47^ and decreased use of COPD medications, hospitalizations, HCRU, and exacerbations following 6-months of initiating nebulized GLY, compared with the pre-index period (6-months prior to initiation)^48^. COPD is prevalent in ~20% of long-term care (LTC) residents^49^.

Patients with COPD in LTC settings often lack the optimal peak inspiratory flow rates for handheld inhalers and may have cognitive/physical impairments that increase device errors^50^. As GLY is delivered via tidal breathing, a coordinated breathing effort during administration is not required; therefore, device handling errors are reduced among elderly patients with COPD. To expand on these studies and guide treatment decisions, it is important to also examine the efficacy of nebulized GLY in additional clinically relevant subgroups of patients with COPD (e.g., current vs. former smokers, FEV_1_ reversibility at baseline, FEV_1_ %predicted ≥80%, LTC). Secondary analyses of Glycopyrrolate Effect on syMptoms and lung function 1 and 2 (GEM1 and GEM2) trials of GLY 15.6 µg BID via Neohaler^®^ DPI in patients with moderate-to-severe COPD show significantly improved lung function and PROs with GLY/Neohaler^®^ compared with placebo, regardless of FEV_1_ reversibility or smoking status at baseline^51,52^. It will be important to determine whether nebulized GLY will offer similar benefits in these subgroups of patients with COPD.

COPD is increasingly being recognized as a multicomponent disorder, and its severity can increase due to comorbidities. The use of a simple comorbidity count method in patients from the GOLDEN 3 and 4 studies showed similarities in the prevalence of comorbidities between these studies and two large cohorts of COPD patients (COPDGene^®^, and SPIROMICS)^33^. In this analysis, nebulized GLY improved lung function and health status in patients irrespective of comorbidity count, supporting the use of nebulized GLY among patients with COPD and comorbidities in clinical practice. To bridge the gap between patients in RCTs and those in the clinic, RCTs should be complemented with real-world observational studies using regional/national COPD databases. Clinical trials with less-stringent inclusion criteria and more representative of patients in clinical practice (e.g., patients with comorbidities, non-adherence, polypharmacy), are also needed to comprehensively understand therapy effectiveness and translate the results of such trials into the clinic.

This article is limited by being an unsystematic review, although the small number of secondary analyses of nebulized GLY from the Phase III trials ensure that we have included all available content. The secondary analyses described here are limited by the post hoc nature of the patient stratification and lack of adjustment for multiplicity. Differences in patient characteristics at baseline may have contributed to some of the observed differences in treatment responses. In addition, although the GOLDEN 3 and 4 studies allowed the inclusion of patients with comorbidities, it must be noted that patients with severe comorbidities (e.g., unstable CVD and/or long QT syndrome) were excluded^7^. Therefore, the study populations may not represent an accurate and complete snapshot of real-world patients with COPD. In addition, large differences in the number of patients within some subgroups (e.g., MetS, A/D, CVD)^34,40,42^, and errors in self-reporting of certain comorbidities (e.g., A/D, SGRQ-based definitions of CB)^42,44^, may have contributed to differential treatment effects observed with nebulized GLY. Additional analyses of clinical trials data by race or ethnicity are necessary but are not feasible with the GOLDEN 3 and 4 studies due to the majority Caucasian patient population; upcoming real-world analyses of nebulized GLY should focus on determining any impact of race or ethnicity on treatment efficacy or safety. Further, greater insights from real-world studies of nebulized GLY, taking into account disease severity, are needed to complement the analyses performed from the GOLDEN 3 and 4 data.

## Conclusions

The GOLDEN 3 and 4 studies demonstrated the efficacy and safety of GLY 25 µg delivered via the eFlow^®^ CS nebulizer in patients with moderate-to-very-severe COPD. Subgroup analyses from these trials have shown that nebulized GLY improved lung function and health status in patients with COPD, regardless of their age, gender, disease characteristics (e.g., disease severity, CB), or comorbidities at baseline; these analyses suggest that nebulized GLY is a safe and effective treatment in patients representative of a real-world COPD population. Further, real-world data show that nebulized GLY decreased hospitalizations and exacerbations following treatment initiation, in the 6-month follow-up period, compared with the 6-month pre-index period, and patients reported a high degree of treatment satisfaction. Together, these results reaffirm that nebulized GLY is an effective therapeutic option for patients with COPD in real-world settings. The insights obtained from these secondary analyses highlight the need for the assessment of future clinical trials of new investigational agents by such clinical variables to ensure efficacy and safety within different patient subpopulations.

### Reporting summary

Further information on research design is available in the Nature Research Reporting Summary linked to this article.

## Supplementary information

Supplementary Information

Reporting Summary

## Data Availability

Sunovion Pharmaceuticals Inc. is part of a clinical trial data-sharing consortium that facilitates access for qualified researchers to selected anonymized clinical trial data. For up-to-date information on data availability, please visit: https://www.clinicalstudydatarequest.com/Study-Sponsors.aspx and click on Sunovion.
