# Supplementary material for: Impact of baseline clinical features on outcomes of nebulized glycopyrrolate therapy in COPD
Source: NPJ Prim Care Respir Med. 2021 Oct 7;31:43. doi: 10.1038/s41533-021-00255-7 (PMC8497491; doi:10.1038/s41533-021-00255-7)
Supplement: Supplementary file 1 — Supplementary Information [file 41533_2021_255_MOESM1_ESM.pdf]

Supplementary Fig. 1 EXACT-RS Responder rates with GLY compared with placebo by **A** baseline demographics and disease severity and **B** comorbidities and chronic bronchitis at baseline.

Notes: *$p < 0.05$ versus placebo.

CI confidence interval, EXACT-RS Exacerbations of Chronic Pulmonary Disease Tool-Respiratory Symptoms, GLY nebulized glycopyrrolate, OR odds ratio, Q quartile.

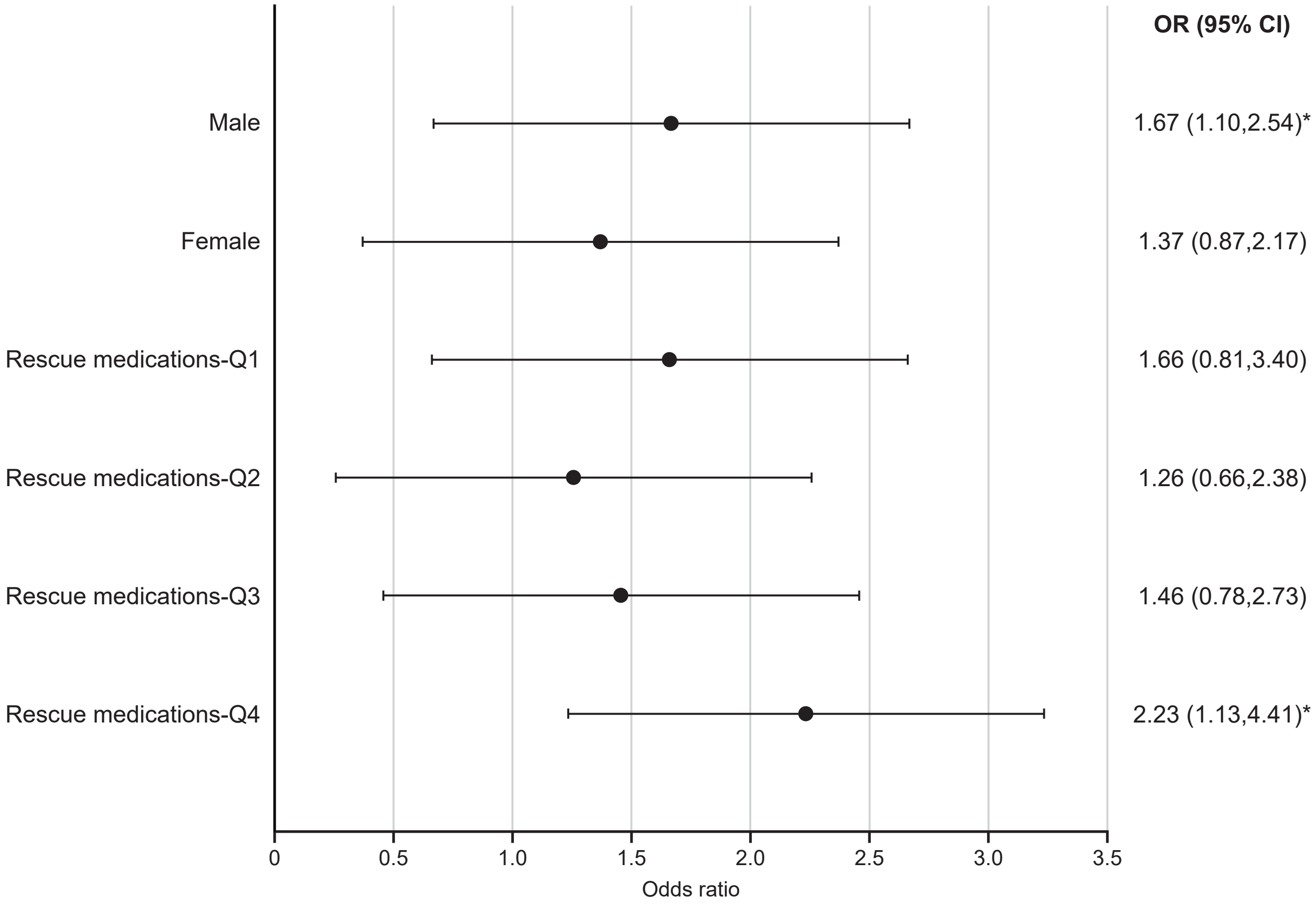
OR (95% CI)
Male
1.67 (1.10,2.54)*
Female
1.37 (0.87,2.17)
Rescue medications-Q1
1.66 (0.81,3.40)
Rescue medications-Q2
1.26 (0.66,2.38)
Rescue medications-Q3
1.46 (0.78,2.73)
Rescue medications-Q4
2.23 (1.13,4.41)*
0
0.5
1.0
1.5
2.0
2.5
3.0
3.5
Odds ratio
